# Application of Computational Intelligence Methods for the Automated Identification of Paper-Ink Samples Based on LIBS

**DOI:** 10.3390/s18113670

**Published:** 2018-10-29

**Authors:** Krzysztof Rzecki, Tomasz Sośnicki, Mateusz Baran, Michał Niedźwiecki, Małgorzata Król, Tomasz Łojewski, U Rajendra Acharya, Özal Yildirim, Paweł Pławiak

**Affiliations:** 1 Faculty of Physics, Mathematics and Computer Science, Cracow University of Technology, Warszawska 24, 31-155 Krakow, Poland; krz@pk.edu.pl (K.R.); tsosnicki@pk.edu.pl (T.S.); mbaran@pk.edu.pl (M.B.); nkg@pk.edu.pl (M.N.); 2 Laboratory for Forensic Chemistry, Faculty of Chemistry, Jagiellonian University, Gronostajowa 2, 30-387 Krakow, Poland; krolm@chemia.uj.edu.pl; 3 Faculty of Materials Science and Ceramics, AGH University of Science and Technology, Mickiewicza 30 Av., 30-059 Krakow, Poland; lojewski@agh.edu.pl; 4 Department of Electronics and Computer Engineering, Ngee Ann Polytechnic, 535 Clementi Rd, 599489 Singapore, Singapore; aru@np.edu.sg; 5 Department of Biomedical Engineering, School of Science and Technology, Singapore School of Social Sciences, 599494 Singapore, Singapore; 6 School of Medicine, Faculty of Health and Medical Sciences, Taylor’s University, 47500 Subang Jaya, Malaysia; 7 Department of Computer Engineering, Munzur University, 62000 Tunceli, Turkey; oyildirim@munzur.edu.tr

**Keywords:** classification, computational intelligence methods, discrimination power, LIBS, machine learning, paper-ink analysis

## Abstract

Laser-induced breakdown spectroscopy (LIBS) is an important analysis technique with applications in many industrial branches and fields of scientific research. Nowadays, the advantages of LIBS are impaired by the main drawback in the interpretation of obtained spectra and identification of observed spectral lines. This procedure is highly time-consuming since it is essentially based on the comparison of lines present in the spectrum with the literature database. This paper proposes the use of various computational intelligence methods to develop a reliable and fast classification of quasi-destructively acquired LIBS spectra into a set of predefined classes. We focus on a specific problem of classification of paper-ink samples into 30 separate, predefined classes. For each of 30 classes (10 pens of each of 5 ink types combined with 10 sheets of 5 paper types plus empty pages), 100 LIBS spectra are collected. Four variants of preprocessing, seven classifiers (decision trees, random forest, *k*-nearest neighbor, support vector machine, probabilistic neural network, multi-layer perceptron, and generalized regression neural network), 5-fold stratified cross-validation, and a test on an independent set (for methods evaluation) scenarios are employed. Our developed system yielded an accuracy of 99.08%, obtained using the random forest classifier. Our results clearly demonstrates that machine learning methods can be used to identify the paper-ink samples based on LIBS reliably at a faster rate.

## 1. Introduction

During the last decade, there have been important developments in laser induced breakdown spectroscopy (LIBS). This atomic emission spectroscopy technique, also known as laser induced plasma spectroscopy (LIPS), is used for qualitative and quantitative chemical analysis of samples in all states of matter [1,2]. In this technique, high-power, short-duration laser pulse causes an ablation of the analyzed material, which due to its high temperature (10,000 K) dissociates into excited ions and atoms. When plasma cloud cools down this excited species revert to lower energy states and emit optical radiation which can be recorded and analyzed, revealing information about the elemental composition of the sample [2]. LIBS spectra are generally very rich in emission lines coming from excited atoms and ions occurring in a high temperature plasma cloud. Physical and chemical phenomena behind LIBS are not fully understood yet, as they are very complex in nature [3,4]. Nevertheless, LIBS applications have recently been rapidly growing due to the number of advantages of this method. The most important ones are minimally destructive measurements with little or no sample preparation, no efficiency, or no possibility to analyze in real-time all elements in a single laser shot in the samples of all three states of matter [5]. For solids, both mapping (2D) and depth profiling (3D) can be obtained. LIBS can be used for quantitative chemical analysis, material identification, and discrimination [1]. This method can be applied in laboratories and industrial plants, and even at stand-off distances of tens of meters [6]. These properties predispose the LIBS technique for use in many fields: space exploration [7], remote analysis of hazardous materials [8], on-line quality control in various industries [9], cultural heritage studies [10], forensic chemistry [11,12], geology [13], weld quality assurance [14], robotics [15], and many others [16,17,18,19].

Although currently LIBS is already an established technology, spectrochemical LIBS analysis is not straightforward [20]. Identification of elemental constituents in the sample is usually based on the strongest lines present in the LIBS spectrum (so called persistent lines [1]), which are compared with a literature database collected for all the elements from the periodic table. This analysis is cumbersome and time-consuming because the emission spectrum is determined also by the properties of the plasma, not only by the composition of the examined sample [21].

At present, the great advantages of LIBS seem to be impaired to some extent by the main drawbacks: problems coming from often poor reproducibility, the impact of sample composition on spectra recorded for individual components (matrix effects), and the difficulty of performing an overall reliable data analysis (sometimes over 500 spectral lines need to be interpreted). Thus, a question is immediately raised as to whether the LIBS method can be supported by the modern achievements in the field of computational intelligence [22] in an attempt to overcome the limitations of the current LIBS spectrum analysis methodology. The analogous problem of classification of a one-dimensional series of data is well known in computer science. The methods developed for this problem have been successfully applied in many areas [23,24,25,26,27,28,29,30,31]. Therefore, the application of machine learning methods to LIBS spectra of samples may certainly give a strong impact for further developments and applications.

Motivated by the aforementioned arguments, we decided to apply computational intelligence methods to the classification of paper-ink samples for forensic purposes, based on LIBS spectra of the samples. The problem of discrimination of different paper-ink samples has already been addressed in previous studies based on the LIBS spectrum analysis. Trejos et al. showed [32] that the highest discrimination power (*DP*) (96.4%) was obtained when comparisons were done qualitatively by spectral overlap of the regions of interest (three different emission lines monitored per element) and quantitatively followed by pairwise comparisons (one emission line per element) using ANOVA (analysis of variance). Kula et al. [33] presented the LIBS method as a useful tool in qualitative elemental differentiation of ink samples. They obtained the discrimination power (*DP*) coefficient of 61%, 82%, and 83% for red, black, and blue inks, respectively. Elsherbiny and Nassef [34] studied the dependence of the obtained spectra of various black gel inks on the wavelength of laser excitation, reporting the DP in the range of 88–91%. In the next study [35], the elemental analysis with the use of LIBS was performed with inkjet inks and toners on office paper. The LIBS results supported by pairwise comparison analysis (ANOVA with Tukey’s post hoc test) provided discrimination power of 98.4% (3 indistinguishable/190 compared pairs) for the toners and 100% for the inkjet inks. Moreover, these three undifferentiated toner pairs were discriminated using a Student’s *t*-test at a 95% confidence limit. The authors claimed that LIBS as a tool for the determination of elemental composition of sample can be a part of a procedure for questioned document examination.

The problem of paper-ink classification, on the other hand, is less common in the literature. To the best of authors’ knowledge there are only two articles dealing with LIBS spectra of paper-ink samples employed in classification/fitting problems by means of computational intelligence techniques. In a paper by Hoehses et al. [36], the benefit of applying several independent chemometric methods to LIBS data was demonstrated. A consecutive methodology of applying soft independent modeling of class analogy (SIMCA) and partial least-squares discriminant analysis (PLS-DA) enabled the step-wise classification of data and separation of inks that were not identified by principal component analysis (PCA). The support vector machine (SVM) yielded a correct classification rate of 87%, and cross-validation accuracy amounted to 81%. In the second paper [37], multiple methods such as three comparative functions (linear correlation, overlapping integral, and sum of squared deviations) and two advanced statistical methods (multivariate curve resolution alternating least squares (MCR-ALS) with classification tree and discriminant analysis (DA)) were applied to statistically evaluate LIBS spectra. The newly introduced MCR-ALS/DA methodology showed identification of the paper and printer type with an accuracy of 96.3% and 83.3%, respectively. Computational intelligence methods analyzed in this study have a larger hypothesis space than SIMCA, PLS-DA, MCR-ALS, or SVM; therefore, they can fit the data more accurately.

In the present paper, we show that the similar problem can be solved using computational intelligence methods. The classification problem discussed in this paper is very difficult due to similarities between LIBS spectra from different paper-ink samples and differences between such spectra from one single class. Additionally, we operate over spectra with a large number of lines with significant noise. To solve this classification problem, we tested many preprocessing ways and computational intelligence methods, but the paper presents only selected and best ones.

## 2. Materials and Methods

### 2.1. Materials

Fifty ballpoint pens (10 items of each of 5 models) produced by four different manufacturers from Germany and Poland were purchased in Poland: Bic Orange fine blue (denoted B), Rystor Kropka 0.5 1986 (denoted R), Staedtler Stick 430F (denoted S), Staedtler Ball 432F (denoted SB), and Toma Sunny fine 050 (denoted T). Fifty sheets (10 sheets of each of 5 types) of five Canadian certified reference papers were used (papers denoted A, D, L, N, and O from the set “Fillers in paper” supplied by A.S.O. Design Canada).

Each particular ink from each of 10 pens of 5 types was deposited (as straight lines) on each of 10 sheets of 5 types of papers, using standard hand pressure. All 2500 (50 sheets of papers with 50 deposits each) paper-ink samples were placed in plastic bags and stored in darkness at room temperature.

In the experiment, data for 30 classes (A, A + B, A + R, A + S, A + SB, A + T, D, D + B, …, O + SB, O + T) based on the combination of ink, paper, and empty papers were recorded. They are summarized in Table 1.

### 2.2. LIBS

The analysis of all paper-ink samples was carried out using a laser induced breakdown spectroscopy system LIBS-6 (Applied Photonics, Skipton North Yorkshire, UK). It consists of an integrated Q-switched Quantel Ultra Nd-YAG laser (Quantel, Les Ulis, France) working at λ = 1064 nm emitting a maximum energy equal to 150 mJ due to one laser pulse (6 ns), and an Avaspec-2048-2-USB2 fibre optic Czerny–Turner spectrometer (6-channel) with a CCD detector (Avantes, NS Apeldoorn, The Netherlands). The system was also equipped with a camera enabled to observe the analyzed object and a movable sample table. The LIBS-6 system was operated by LIBSoft V6.0.1 software (Applied Photonics, Skipton North Yorkshire, UK). Under normal conditions, because of no moving elements inside, a wavelength calibration of the spectrometer was not required. Every measurement were conducted in air under atmospheric pressure. The spectrum of a new background was collected prior to the analysis of each new sample.

A Q-switch delay time of 165 μs, the integration delay time of 1.27 μs, and integration time of 1.2 ms were used. Samples were analyzed directly without any special preparation. They were placed at the sample table at the focal point of the focusing lens (at a distance determined by the nozzle about 70 mm from the optical head). The diameter of the ablation spot, ranging from 0.6 mm to 1 mm, was dependent on the analyzed material.

The results of LIBS analysis is an emission spectrum—a two-dimensional dependence between the intensity and the wavelength (nm) of electromagnetic radiation emitted by an atom or molecule making a transition from a high energy state to a lower energy state. There are many possible electron transitions for each atom, and each has a specific energy difference. These different transitions, leading to different radiated wavelengths, create a unique emission spectrum. The emission spectra were collected in the UV-vis range (185 nm to 904 nm, spectral resolution 0.1 nm).

**Figure 1:**
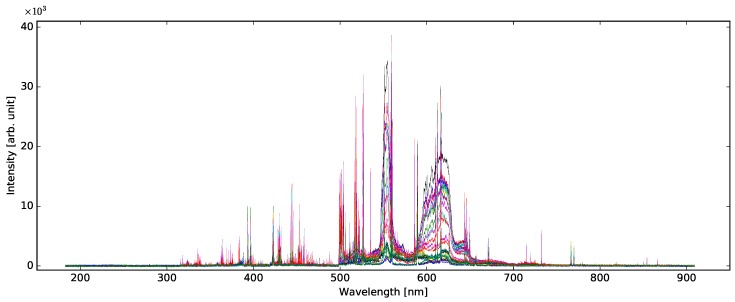
First spectrum of each class.

In total, 30 classes of experimental data listed above were recorded. For each class, 100 LIBS spectra were acquired, resulting in total in 3000 LIBS emission spectra, each one constituting a single sample. There was one spectrum collected from each line on a piece of paper. A sample spectrum is shown in Figure 1. A detailed version of Figure 1 with descriptions of the most prominent lines is in Supplementary Materials. The spectrum of each sample is available on our website [38].

### 2.3. Spectral Line Identification

In the spectral line identification process (Table 2), the standard protocol was applied [1]. Different elemental compositions for different types of papers and inks were identified. In order to find which spectral line in a particular spectrum of a paper-ink sample originates from ink, a comparison with a spectrum of pure paper was made. The general observation was that the elemental profile of all papers is in accordance with the information (included in the certificate of analysis) about fillers used in analyzed standard papers. Thus, the identified elements were mainly corresponding to the following fillers: *calcium carbonate* $\left({\mathrm{CaCO}}_{3}\right)$, *kaolinite* $\left({\mathrm{Al}}_{4}\left[{\mathrm{Si}}_{4}O_{10}\right]\left({\mathrm{OH}}_{8}\right)\right)$, *titanium dioxide* $\left({\mathrm{TiO}}_{2}\right)$, and *talc* $\left({\mathrm{Mg}}_{3}{\left(\mathrm{OH}\right)}_{2}{\mathrm{Si}}_{4}O_{10}\right)$. In spectra obtained for papers containing ${\mathrm{TiO}}_{2}$ (L and N) due to the ubiquity of Ti spectral lines, no lines from ink components were observable. The best searching of ink spectral lines of elements originating from inks was in the case of O paper in which kaolinite was the main filler. Concerning ballpoint pen inks, four of them have relatively complicated elemental composition (at least three elements). In one of them (ink T) only one element was found.

### 2.4. Data Analysis

We first performed a visual inspection of data after using PCA. In Supplementary Materials, we include 14 figures presenting data after PCA for all classes on truncated and normalized data. As can be seen, various types of paper (A, D, L, N, and O) separated well. In contrast, various types of inks (B, R, S, SB, and T) were not distinct from each other. This visual analysis confirms the difficulty of the issue—the automated identification of paper-ink samples.

The analysis of the LIBS spectrum consisted of the following steps:

1. independent preprocessing of LIBS spectra;
2. selection of data for the cross validation and testing sets;
3. data analysis based on computational intelligence methods;
4. evaluation of the results.

**Figure 2:**
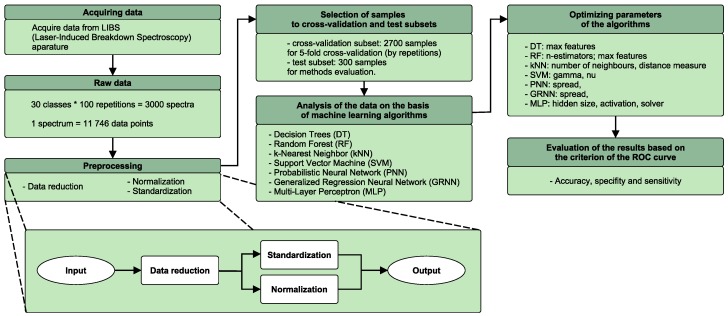
Flow chart of the experiment.

Steps of the experiment are presented in the flowchart in Figure 2.

#### 2.4.1. Signal Preprocessing

The initial preprocessing of the raw data was performed to reduce the number of data points corresponding to individual LIBS spectra. This preprocessing step was applied because the spectral range of LIBS is very broad (11,746 data points in this case). From the viewpoint of computational intelligence applied to classification tasks, it contains irrelevant information. Moreover, the removed spectral ranges are also not chemically relevant—there is only noise (the intensities close to zero) and there are no spectral lines. Therefore, these ranges do not provide any relevant information about the elemental composition of the sample. It can be expected that the truncation of the datasets enhances extraction of characteristic features of the distinctive classes (types of material) [39]. Four preprocessing steps were taken under consideration:

- data truncation by removing the data points from the beginning (first 3746 data points) and from the end (last 1000 data points) of the LIBS spectrum, which do not contain relevant information (7000 data points are left for further analysis);
- normalization of intensity values to the interval [0,1];
- standardization of intensity values (therefore, the mean value becomes equal to 0 and standard deviation becomes equal to 1).

Four preprocessing ways based on these steps were constructed and evaluated: data truncation and either standardization (also known as standard normal variate) or normalization. These steps are depicted at the bottom of Figure 2.

The Simple Intuitive Language for Experiment Modeling (SILEM) [40] software was used for data preprocessing tasks.

#### 2.4.2. Cross-Validation

The set of 3000 LIBS emission spectra was divided into two subsets [41]: a training subset containing 90% (2700) of spectra and a test subset containing 10% (300) of spectra. Selection of spectra for both subsets was performed in a stratified way, that is the numbers of spectra from different classes had the same proportions in each subset. The cross-validation subset was used for parameter optimization, and the test subset was then used for final evaluation of different types of classifiers with optimized parameters.

The 5-fold stratified cross-validation method was applied to build the training and validation data sets. In each of the five analyzed combinations, a training set of 72 LIBS spectra from each of 30 classes (2160 in total) and a validation set of 18 LIBS spectra from each of 30 classes (540 in total) were used.

Evaluation of the classifiers with optimized parameters was performed separately for each training subset using the test subset. Results of evaluation based on each training subset were obtained by averaging the folds.

#### 2.4.3. Computational Intelligence Methods

The LIBS spectra, after preprocessing and cross-validation, were fed to the classifiers: generalized regression neural network (GRNN) [42], probabilistic neural network (PNN) [43], multi-layer perceptron (MLP) [44], support vector machine (SVM) [45], decision trees (DT) [46], *k*-nearest neighbor (kNN) classifier [47], and random forest (RF) [46].

Each method is potentially dependent on a set of parameters that are either quantitative of qualitative. They have an influence on the overall performance of the method. These parameters were separately optimized for each machine learning algorithm to receive the lowest number of erroneous classes. The basic categorical parameters for each method were set to constants listed in Table 3.

Methods based on an adaptive neuro-fuzzy inference system (ANFIS) [48] and Gaussian process [49] were also tested, but they are computationally intensive and take a long time to receive results even with a reduced data set.

The SILEM software uses the scikit-learn Python library [50] as a source of implementations of employed computational intelligence algorithms.

#### 2.4.4. Evaluation Criteria

Evaluation of classification process was based on methodology described in [51,52]. The 5-fold cross-validation strategy was adopted in our experiment. Five performance parameters (figures of merit), namely accuracy (*ACC*), sensitivity (*SEN*), specificity (*SPE*), mean values, and Cohen’s kappa (κ), were calculated. The mean values were calculated to estimate the overall performance of the computational intelligence methods used in this study separately for the task of recognition of each of the different classes of LIBS spectra.

To test whether there are classes which are classified with higher accuracy, specificity, or sensitivity, we calculated for each class *S* the number of true positives TP(S), false positives FP(S), true negatives TN(S), and false negatives FN(S) separately. Then, the accuracy ACC(S), sensitivity SEN(S), and specificity SPE(S) with respect to class *S* are defined as averages over five folds of cross-validation (K=5 is the number of folds):

(1) $$\mathrm{ACC}\left(S\right)=\frac{1}{K}\sum_{i=1}^{K}\frac{{\mathrm{TP}}_{i}\left(S\right)+{\mathrm{TN}}_{i}\left(S\right)}{{\mathrm{TP}}_{i}\left(S\right)+{\mathrm{FP}}_{i}\left(S\right)+{\mathrm{TN}}_{i}\left(S\right)+{\mathrm{FN}}_{i}\left(S\right)}$$

(2) $$\mathrm{SEN}\left(S\right)=\frac{1}{K}\sum_{i=1}^{K}\frac{{\mathrm{TP}}_{i}\left(S\right)}{{\mathrm{TP}}_{i}\left(S\right)+{\mathrm{FN}}_{i}\left(S\right)}$$

(3) $$\mathrm{SPE}\left(S\right)=\frac{1}{K}\sum_{i=1}^{K}\frac{{\mathrm{TN}}_{i}\left(S\right)}{{\mathrm{TN}}_{i}\left(S\right)+{\mathrm{FP}}_{i}\left(S\right)}$$

where ${TP}_{i}\left(S\right)$, ${\mathrm{FP}}_{i}\left(S\right)$, ${\mathrm{TN}}_{i}\left(S\right)$, and ${\mathrm{FN}}_{i}\left(S\right)$ are, respectively, the number of true positives, false positives, true negatives, and false negatives for the *i*th fold of cross-validation with respect to class *S*, i=1,2,…,K.

The overall values of ACC, SEN, and SPE for the classification system are the arithmetic means of ACC(S), SEN(S), and SPE(S) over all classes.

To evaluate the degree of discrimination between two different samples discrimination power (also known as discrimination accuracy) coefficient (denoted *DP*) was proposed [53,54]. *DP* is the ratio of the number of correctly identified pairs of test samples (identified as from different classes) to the number of all possible pairs of test samples. It can be calculated by the following equation:

(4) $$\mathrm{DP}=\frac{2D}{T(T-1)}=1-\frac{2N}{T(T-1)}$$

where

- *D*—the number of differentiated pairs, that is the number of pairs correctly identified as belonging to the same class or correctly identified as belonging to different classes;
- *N*—the number of non-differentiated pairs, N=T(T−1)/2−D;
- *T*—the total number of analyzed samples (the total number of possible pairs of samples is equal to T(T−1)/2).

## 3. Results

**Figure 3:**
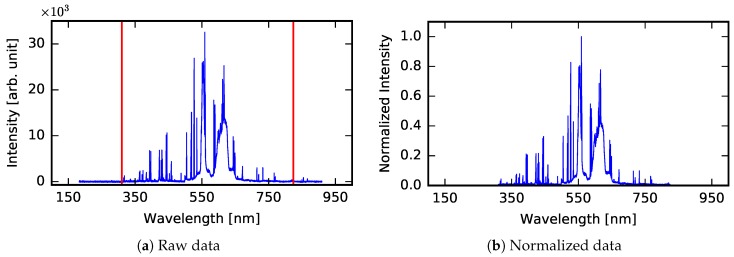
Visualization of data preprocessing for single sample spectrum from Class A. Figure (**a**) depicts an example of a raw spectrogram with marked truncation points, (**b**) shows normalized data.

An example of a raw spectrum and the output from successive stages of spectrum preprocessing (data truncation and normalization) are shown in Figure 3. The data truncation stage is optional and normalization can be replaced by standardization.

Parameter selection is a key part in reaching the optimal overall performance of a classification system. There are many possible options available, so it is important to analyze the outcomes of experiments for different values of parameters to demonstrate the possibilities of machine learning methods. Particular classification methods depend on the various basic parameters set as listed in Table 3 and some other parameters that were optimized. Optimization of these parameters was performed in two steps. The first step was to find out the general range of values for each parameter for the fine-tuning procedure. Then, the detailed grid search of the selected ranges of parameters was performed.

The classification algorithms, the tuning parameters and range of these parameter values are described below.

- Decision trees—the range of the number of features to consider when looking for the best split: from 100 to 7000 with a step equal to 100.
- Random forest—two parameters were optimized during preprocessing. The number of trees in the forest was optimized in range from 10 to 200 with a step of 10 and from 200 to 1000 with a step equal to 50, and the number of features to consider when looking for the best split was optimized in the same range.
- *k*NN—the number of neighbors was optimized in the range from 1 to 4 and exponent used to calculate the Minkowski distance was optimized from 1 to 10 with a step of 1.
- SVM—the gamma parameter of the RBF kernel function was optimized in a range from 0.01 to 1.00 with a step of 0.01 and the nu parameter of the nu-SVC algorithm, related to the error tolerance of the SVM classification, was optimized in a range from 0.01 to 1.00 with a step of 0.01.
- PNN—the radius (the spread) of the kernel function of the network (standard deviation for the probability density function of the normal distribution). This parameter was optimized in a range from 0.01 to 0.20 with a step equal to 0.01 when normalization in preprocessing was used and from 0.1 to 1.0 with a step of 0.1 when standardization was used.
- GRNN—the spread parameter with an identical meaning and range as in PNN was optimized;
- MLP—the number of neurons was optimized in a range from 10 to 200 with a step equal to 10. The activation function was selected from “identity,” “logistic,” “tanh,” “relu”. The solver for weight optimization was chosen from “lbfgs” (an optimizer in the family of quasi-Newton methods), “sgd” (a stochastic gradient descent), or “adam” (a stochastic gradient-based optimizer proposed in [55]).

These optimal parameters were selected in a series of experiments to maximize the mean *ACC*, *SEN*, and *SPE* values described in Section 2.4.4.

The best results of the experiments with different preprocessing methods and machine learning algorithms based on 5-fold cross-validation (3000 spectra were divided into training set containing 2160 spectra, a validation set with 540 spectra, and test sets containing 300) are presented in Table 4. The preprocessing involves data truncation and standardization or normalization. Generally, the classification results were worse when the preprocessing included normalization instead of standardization. In the table, we show the values of *ACC*, *SEN*, *SPE* (and their mean value denoted *MEAN*), κ, and *DP*.

Table 5 shows the values of ACC(S), SEN(S), and SPE(S) for all *S* classes and all classifiers with appropriate preprocessing methods. It can be seen from the table that the most easily distinguishable (highest ACC(S), SPE(S), and SEN(S)) spectra are in six classes: A, A + T, D, N, O, and O + T (ACC(S), SPE(S), and SEN(S) reached the value of 100%) using the random forest method. Additionally, spectra of samples from 2 classes—L and N + T—are well recognized by SVM and MLP methods as well. The least distinguishable classes are L + S and O + B with a value of SEN(S) less than 50% by the random forest method, but the SPE(S) value was over 99%. This means that the spectra of samples from these classes are often assigned to other classes.

## 4. Discussion

The results of experiments confirm that the computational intelligence methods can be used to analyze LIBS data and obtain accurate classification of paper-ink samples (please see Table 4). We have obtained an accuracy of 99.08% for the best classifier. The results of the present study cannot be directly compared with those obtained in [33,34] focusing on the problem of discrimination between two samples which were not assigned to a priori classes. However, the methods used in [33,34] used Plasus SpecLine 2.13 software for the identification of spectral lines and National Institute of Standards and Technology (NIST) spectral database, gave values of the *DP* in the range from 85 to 92%. The same standard methods with visual spectra was applied to the data collected in our study gave *DP* equal to 90.6%. After adopting our algorithms to the problem of discrimination between two different samples, we achieved the *DP* of 99.0% with SVM and more than 98% with decision trees, random forest, PNN, and MLP classifiers. The limitation of applying our solution to the problem of discrimination is that the classes must be predefined.

The detailed analysis of Table 4 shows that the best method for the class of problems addressed in the present study is the random forest classifier. Parameters for this classifier were optimized using the 5-fold cross-validation using standardized data. The classifier during test over independent test set was able to correctly recognize 1294 cases of LIBS spectra out of 1500 cases and had no erroneous recognitions in training sets (10,800 cases). The cases are summed across all five folds of cross-validation. This classifier achieved an accuracy of 99.08%, a sensitivity of 86.27%, a specificity of 99.53%, a kappa of 85.79% and a *DP* of 99.08% for the test set.

Taking into account the number of erroneous classifications under different settings, the worst classifier was GRNN.

Two preprocessing methods were applied on the raw data prior to the classification. Hence, we have shown that an appropriate selection of preprocessing procedures influences the performance of classification. However, although in most cases the actual selection of the pair preprocessing-classifier determines the overall performance of the classification method, for some specific selections of classifier the final classification performance depends only marginally on the preprocessing. For this reason, it is not possible to select a single best preprocessing method.

We have taken two other methods into consideration: resampling and wavelet decomposition. The former resamples the input data at given wavelengths of the original sequence. The latter performs one dimensional Haar wavelet decomposition [56]. The initial results were promising, but this method requires more detailed investigation in the future. This technique needs further research to determine which type of wavelet decomposition structure and which parameters will lead to the best results.

The results of the present study show that modern computational intelligence methods can be used for the classification of LIBS spectra into predefined classes, which solves a broad class of problems related to LIBS applications. The main limitation of this study is that with the chosen settings, another broad class of LIBS applications, that is analysis of elemental composition, cannot be solved. The problem of classification addressed in the present study is certainly simpler than the problem of detailed analysis of the composition of materials. However, a successful solution to this problem encourages further research in this area. We suppose that, because the problem of analysis of composition of materials based on a LIBS spectrum may be equivalent to the recognition of a presence of specific spectral lines, this problem can be brought to a series of classification subproblems.

## 5. Conclusions

The aim of this study was to solve the difficult problem of distinguishing paper-ink samples. Hence, we have designed a computational intelligence system to solve this problem using LIBS spectra. The difficulty of this problem is caused by the high variability of spectra within a single class of paper-ink samples and strong similarity of spectra from samples of different classes.

A machine learning system is developed and based on results presented in Table 4 and Table 5, we can conclude that the system performs well. The random forest classifier provided the most accurate classification with an accuracy of 99.08%, a sensitivity of 86.27%, a specificity of 99.53%, a kappa of 85.79% and a *DP* of 99.08% for the test set.

Our developed system can be used for other applications, e.g., the identification of metal alloys. The main limitation of this method is that we do not list particular elements of the tested material, but it may be considered as future work. Additional feature extraction and classification methods, such as principal component analysis [57], can be applied in the future. Hence, the sensitivity of the described method can also be further improved.

## Figures and Tables

**Table 1:** List of papers and ballpoint pens examined in this study.

Pens		Papers				
Company/Model	ID	A	D	L	N	O
Lack	–	A	D	L	N	O
Bic	B	A + B	D + B	L + B	N + B	O + B
Rystor	R	A + R	D + R	L+R	N + R	O + R
Staedtler/Stick	S	A + S	D + S	L + S	N + S	O + S
Staedtler/Ball	SB	A + SB	D + SB	L + SB	N + SB	O + SB
Toma	T	A + T	D + T	L + T	N + T	O + T

**Table 2:** Spectral line identification.

Paper Class	Identified Elements
A	Ca, Mg, Na, K
D	Ca, Na, K
L	Ca, Ti, Al, Si, Na, K
N	Ca, Ti, Si, Mg, Fe, Na, K
O	Ca, Al, Si, Mg, Na, K
**Ink sample**	
B	Cr, Cu, Zn, Pb, La
R	Cr, Cu, Zn, Pb, Ni, Mn
S	Cr, Cu, Zn
SB	Cr, Cu, Zn, Pb
T	Cr

**Table 3:** Computational intelligence methods and their basic parameters used for LIBS spectra identification.

No.	Method	Configuration
**1**	Decision Trees	Criterion: gini, splitter type: best, maximum depth: none
**2**	Random Forest	Criterion: gini, maximum depth: none
**3**	kNN	Distance metric: Minkowski
**4**	SVM	Type: nuSVC, type of kernel function: radial basis function
**5**	Neural Network	Type: PNN
**6**	Neural Network	Type: GRNN
**7**	Neural Network	Type: MLP

**Table 4:** Performance results for optimized parameters.

Method	Parameters	ACC (%)	SEN (%)	SPE (%)	MEAN (%)	κ (%)	DP (%)
Decision Trees	Max features = 6100	98.08	71.13	99.00	89.40	70.14	98.40
Random Forest	N estimators = 700 Max features = 950	99.08	86.27	99.53	94.96	85.79	99.08
kNN	N = 1 Exponent = 1.00	96.72	50.87	98.31	81.97	49.17	96.72
SVM	Nu = 0.17 Gamma = 0.03	98.84	82.53	99.40	93.59	81.93	98.85
PNN	Spread = 0.2	96.84	52.60	98.37	82.60	50.97	97.80
GRNN	Spread = 0.5	96.27	44.00	98.07	79.45	42.07	95.58
MLP	N. of neurons = 120	98.22	73.27	99.08	90.19	72.34	98.36

**Table 5:** Results of classification per class.

	Standardization Decision Tree			Standardization Random Forest			Standardization kNN			Normalization SVM			Normalization PNN			Standardization GRNN			Standardization MLP		
Class	ACC(%)	SPE(%)	SEN(%)	ACC(%)	SPE(%)	SEN(%)	ACC(%)	SPE(%)	SEN(%)	ACC(%)	SPE(%)	SEN(%)	ACC(%)	SPE(%)	SEN(%)	ACC(%)	SPE(%)	SEN(%)	ACC(%)	SPE(%)	SEN(%)
A	99.20	99.45	92.00	100.00	100.00	100.00	100.00	100.00	100.00	100.00	100.00	100.00	99.40	99.38	100.00	99.73	99.72	100.00	99.93	99.93	100.00
A + B	98.60	99.45	74.00	99.33	100.00	80.00	97.53	98.97	56.00	99.27	99.86	82.00	97.40	99.17	46.00	98.53	99.59	68.00	98.60	99.59	70.00
A + R	97.93	99.24	60.00	99.40	99.72	90.00	97.87	99.66	46.00	99.33	100.00	80.00	97.40	99.38	40.00	98.27	99.45	64.00	97.87	98.83	70.00
A + S	96.60	97.93	58.00	99.40	99.72	90.00	96.80	97.86	66.00	99.33	99.72	88.00	96.47	98.69	32.00	97.27	98.07	74.00	96.87	97.86	68.00
A + SB	96.40	97.93	52.00	98.67	98.90	92.00	97.20	98.14	70.00	98.60	98.83	92.00	95.27	95.93	76.00	97.47	98.90	56.00	96.80	98.69	42.00
A + T	98.80	99.72	72.00	100.00	100.00	100.00	99.67	99.72	98.00	99.60	99.59	100.00	98.33	99.31	70.00	99.27	99.38	96.00	99.67	99.72	98.00
D	99.80	99.79	100.00	100.00	100.00	100.00	98.60	98.55	100.00	99.87	99.93	98.00	98.47	98.76	90.00	99.33	99.31	100.00	99.87	99.86	100.00
D + B	98.07	99.03	70.00	99.33	99.72	88.00	95.80	98.48	18.00	98.47	99.59	66.00	95.67	98.28	20.00	96.27	99.31	8.00	95.73	98.28	22.00
D + R	98.40	99.17	76.00	99.93	99.93	100.00	97.80	99.79	40.00	99.73	99.72	100.00	96.67	98.28	50.00	98.20	99.31	66.00	97.93	99.52	52.00
D + S	98.53	99.17	80.00	99.53	100.00	86.00	96.40	97.17	74.00	99.53	99.86	90.00	95.40	97.66	30.00	97.13	97.66	82.00	95.60	97.10	52.00
D + SB	96.67	98.21	52.00	98.80	99.59	76.00	96.07	98.14	36.00	97.73	98.76	68.00	94.60	97.31	16.00	97.40	98.55	64.00	95.73	97.86	34.00
D + T	99.13	99.59	86.00	99.07	99.03	100.00	98.40	99.10	78.00	99.20	99.31	96.00	97.20	98.34	64.00	98.33	98.97	80.00	99.00	99.10	96.00
L	99.40	99.45	98.00	99.53	99.52	100.00	96.27	96.14	100.00	100.00	100.00	100.00	98.80	98.76	100.00	94.67	94.48	100.00	100.00	100.00	100.00
L + B	98.07	99.24	64.00	99.87	99.93	98.00	96.47	99.38	12.00	99.27	99.59	90.00	95.87	97.59	46.00	96.33	99.59	2.00	98.47	99.59	66.00
L + R	97.73	99.45	48.00	98.47	99.93	56.00	96.80	99.45	20.00	98.20	99.24	68.00	96.87	98.90	38.00	96.33	99.59	2.00	97.93	99.72	46.00
L + S	96.00	98.07	36.00	97.20	99.17	40.00	96.00	98.97	10.00	97.47	99.59	36.00	95.80	98.48	18.00	96.47	99.45	10.00	96.73	98.07	58.00
L + SB	95.67	96.55	70.00	96.67	97.10	84.00	91.53	93.45	36.00	97.00	97.52	82.00	94.87	96.34	52.00	91.20	92.97	40.00	97.27	97.66	86.00
L + T	98.07	99.45	58.00	99.20	99.66	86.00	96.00	98.69	18.00	99.53	99.66	96.00	97.93	99.66	48.00	95.27	98.55	0.00	99.60	99.79	94.00
N	99.87	99.93	98.00	100.00	100.00	100.00	99.07	99.03	100.00	100.00	100.00	100.00	100.00	100.00	100.00	98.33	98.28	100.00	100.00	100.00	100.00
N + B	98.33	99.66	60.00	98.73	99.59	74.00	96.93	99.24	30.00	99.67	100.00	90.00	97.47	98.62	64.00	95.67	98.90	2.00	98.80	99.66	74.00
N + R	98.40	99.59	64.00	99.53	100.00	86.00	95.67	98.97	0.00	97.67	99.45	46.00	95.33	97.86	22.00	95.60	98.90	0.00	98.13	99.59	56.00
N + S	97.60	98.55	70.00	99.27	99.93	80.00	97.60	99.52	42.00	98.13	99.45	60.00	95.80	98.34	22.00	96.87	99.72	14.00	98.47	99.17	78.00
N + SB	96.33	97.59	60.00	97.33	98.00	78.00	92.47	93.93	50.00	96.53	96.97	84.00	94.47	96.76	28.00	88.53	90.14	42.00	96.73	97.52	74.00
N + T	99.33	99.45	96.00	99.67	99.66	100.00	99.07	99.38	90.00	100.00	100.00	100.00	99.07	99.17	96.00	96.60	99.38	16.00	100.00	100.00	100.00
O	99.20	99.31	96.00	100.00	100.00	100.00	99.93	99.93	100.00	100.00	100.00	100.00	100.00	100.00	100.00	99.87	99.86	100.00	100.00	100.00	100.00
O + B	96.73	98.90	34.00	97.53	99.93	28.00	96.60	99.93	0.00	98.00	99.86	44.00	97.20	99.38	34.00	96.33	99.66	0.00	97.80	100.00	34.00
O + R	99.47	99.86	88.00	99.67	100.00	90.00	96.60	99.93	0.00	98.93	99.86	72.00	96.53	98.90	28.00	96.27	99.59	0.00	99.00	100.00	70.00
O + S	98.73	99.45	78.00	99.47	99.86	88.00	96.27	99.45	4.00	98.07	99.24	64.00	95.27	98.00	16.00	96.33	99.66	0.00	98.20	98.83	80.00
O + SB	95.87	97.31	54.00	96.93	96.90	98.00	87.93	89.45	44.00	96.00	96.41	84.00	92.87	94.90	34.00	84.67	86.55	30.00	96.13	96.76	78.00
O + T	99.33	99.66	90.00	100.00	100.00	100.00	98.40	98.76	88.00	99.93	99.93	100.00	98.80	98.83	98.00	95.47	98.62	4.00	99.67	99.66	100.00
Mean	98.08	99.00	71.13	99.08	99.53	86.27	96.72	98.31	50.87	98.84	99.40	82.53	96.84	98.37	52.60	96.27	98.07	44.00	98.22	99.08	73.27
Kappa	70.14			85.79			49.17			81.93			50.97			42.07			72.34		
DP	98.40			99.08			96.72			98.85			97.80			95.58			98.36		

## Supplementary Materials

The following are available online at http://www.mdpi.com/1424-8220/18/11/3670/s1.

## References

[1] Singh J., Thakur S. (2007). Laser-Induced Breakdown Spectroscopy.

[2] Anabitarte F., Cobo A., Lopez-Higuera J.M. (2012). Laser-Induced Breakdown Spectroscopy: Fundamentals, Applications, and Challenges. Int. Sch. Res. Not. Spectrosc..

[3] Hahn D.W., Omenetto N. (2010). Laser-induced breakdown spectroscopy (LIBS), part I: Review of basic diagnostics and plasma-particle interactions: Still-challenging issues within the analytical plasma community. Appl. Spectrosc..

[4] Hahn D.W., Omenetto N. (2012). Laser-induced breakdown spectroscopy (LIBS), part II: Review of instrumental and methodological approaches to material analysis and applications to different fields. Appl. Spectrosc..

[5] Galbács G. (2015). A critical review of recent progress in analytical laser-induced breakdown spectroscopy. Anal. Bioanal. Chem..

[6] Stelmaszczyk K., Rohwetter P., Méjean G., Yu J., Salmon E., Kasparian J., Ackermann R., Wolf J.P., Wöste L. (2004). Long-distance remote laser-induced breakdown spectroscopy using filamentation in air. Appl. Phys. Lett..

[7] Wiens R., Maurice S. The ChemCam investigation: Compositions at the curiosity rover landing site. Proceedings of the 2012 GSA Annual Meeting in Charlotte.

[8] Ramirez-Cedeno M., Ortiz-Rivera W., Pacheco-Londono L., Hernandez-Rivera S. (2010). Remote Detection of Hazardous Liquids Concealed in Glass and Plastic Containers. IEEE Sens. J..

[9] Noyel M., Thomas P., Charpentier P., Thomas A., Brault T. Implantation of an on-line quality process monitoring. Proceedings of the 2013 International Conference on Industrial Engineering and Systems Management (IESM).

[10] Ortiz R., Ortiz P., Colao F., Fantoni R., Gómez-Morón M., Vázquez M. (2015). Laser spectroscopy and imaging applications for the study of cultural heritage murals. Constr. Build. Mater..

[11] Rinke-Kneapler C., Sigman M., Baudelet M. (2014). Applications of laser spectroscopy in forensic science. Laser Spectroscopy for Sensing.

[12] Król M., Kowalska D., Kościelniak P. (2018). Examination of Polish Identity Documents by Laser-Induced Breakdown Spectroscopy. Anal. Lett..

[13] Gaft M., Sapir-Sofer I., Modiano H., Stana R. (2007). Laser induced breakdown spectroscopy for bulk minerals online analyses. Spectrochim. Acta Part B At. Spectrosc..

[14] Anabitarte F., Mirapeix J., Portilla O.M.C., Lopez-Higuera J.M., Cobo A. (2012). Sensor for the Detection of Protective Coating Traces on Boron Steel With Aluminium–Silicon Covering by Means of Laser-Induced Breakdown Spectroscopy and Support Vector Machines. IEEE Sens. J..

[15] Bukin O., Proschenko D., Chekhlenok A., Golik S., Bukin I., Mayor A., Yurchik V. (2018). Laser Spectroscopic Sensors for the Development of Anthropomorphic Robot Sensitivity. Sensors.

[16] Moncayo S., Manzoor S., Navarro-Villoslada F., Caceres J.O. (2015). Evaluation of supervised chemometric methods for sample classification by Laser Induced Breakdown Spectroscopy. Chemom. Intell. Lab. Syst..

[17] Zhang T., Xia D., Tang H., Yang X., Li H. (2016). Classification of steel samples by laser-induced breakdown spectroscopy and random forest. Chemom. Intell. Lab. Syst..

[18] Wang N., Wang X., Chen P., Jia Z., Wang L., Huang R., Lv Q. (2018). Metal Contamination Distribution Detection in High-Voltage Transmission Line Insulators by Laser-induced Breakdown Spectroscopy (LIBS). Sensors.

[19] Zhang C., Shen T., Liu F., He Y. (2018). Identification of Coffee Varieties Using Laser-Induced Breakdown Spectroscopy and Chemometrics. Sensors.

[20] El Haddad J., Canioni L., Bousquet B. (2014). Good practices in LIBS analysis: Review and advices. Spectrochim. Acta Part B At. Spectrosc..

[21] Tognoni E., Palleschi V., Corsi M., Cristoforetti G., Omenetto N., Gornushkin I., Smith B.W., Winefordner J.D. (2006). Laser-Induced Breakdown Spectroscopy (LIBS): Fundamentals and Applications, from Sample to Signal in LIBS.

[22] Tadeusiewicz R. (2010). Place and Role of Intelligent Systems in Computer Science. Comput. Meth. Mater. Sci..

[23] Pławiak P., Sośnicki T., Niedźwiecki M., Tabor Z., Rzecki K. (2016). Hand Body Language Gesture Recognition Based on Signals From Specialized Glove and Machine Learning Algorithms. IEEE Trans. Ind. Inform..

[24] Pławiak P. (2014). An estimation of the state of consumption of a positive displacement pump based on dynamic pressure or vibrations using neural networks. Neurocomputing.

[25] Pławiak P., Maziarz W. (2014). Classification of tea specimens using novel hybrid artificial intelligence methods. Sens. Actuators B Chem..

[26] Rzecki K., Pławiak P., Niedźwiecki M., Sośnicki T., Leśkow J., Ciesielski M. (2017). Person recognition based on touch screen gestures using computational intelligence methods. Inf. Sci..

[27] Pławiak P., Tadeusiewicz R. (2014). Approximation of phenol concentration using novel hybrid computational intelligence methods. Int. J. Appl. Math. Comput. Sci..

[28] Pławiak P., Rzecki K. (2015). Approximation of Phenol Concentration Using Computational Intelligence Methods Based on Signals From the Metal-Oxide Sensor Array. IEEE Sens. J..

[29] Pławiak P. (2018). Novel methodology of cardiac health recognition based on ECG signals and evolutionary-neural system. Expert Syst. Appl..

[30] Pławiak P. (2018). Novel genetic ensembles of classifiers applied to myocardium dysfunction recognition based on ECG signals. Swarm Evol. Comput..

[31] Abdar M., Zomorodi-Moghadam M., Das R., Ting I.H. (2017). Performance analysis of classification algorithms on early detection of liver disease. Expert Syst. Appl..

[32] Trejos T., Flores A., Almirall J.R. (2010). Micro-spectrochemical analysis of document paper and gel inks by laser ablation inductively coupled plasma mass spectrometry and laser induced breakdown spectroscopy. Spectrochim. Acta Part B At. Spectrosc..

[33] Kula A., Wietecha-Posłuszny R., Pasionek K., Król M., Woźniakiewicz M., Kościelniak P. (2014). Application of laser induced breakdown spectroscopy to examination of writing inks for forensic purposes. Sci. Justice.

[34] Elsherbiny N., Nassef O.A. (2015). Wavelength dependence of laser induced breakdown spectroscopy (LIBS) on questioned document investigation. Sci. Justice.

[35] Lennard C., El-Deftar M.M., Robertson J. (2015). Forensic application of laser-induced breakdown spectroscopy for the discrimination of questioned documents. Forensic Sci. Int..

[36] Hoehse M., Paul A., Gornushkin I., Panne U. (2011). Multivariate classification of pigments and inks using combined Raman spectroscopy and LIBS. Anal. Bioanal. Chem..

[37] Metzinger A., Rajkó R., Galbács G. (2014). Discrimination of paper and print types based on their laser induced breakdown spectra. Spectrochim. Acta Part B At. Spectrosc..

[38] Team of Science and Industrial Intelligent Applications. http://siia.iti.pk.edu.pl/.

[39] Zieliński T.P. (2005). Digital Signal Processing: From Theory to Applications.

[40] Simple Intuitive Language for Experiment Modeling. http://silem.iti.pk.edu.pl.

[41] Murphy K.P. (2012). Machine Learning: A Probabilistic Perspective.

[42] Specht D.F. (1991). A general regression neural network. IEEE Trans. Neural Netw..

[43] Specht D.F. (1990). Probabilistic neural networks. Neural Netw..

[44] Hinton G.E. (1989). Connectionist Learning Procedures. Artif. Intell..

[45] Cortes C., Vapnik V. (1995). Support-vector networks. Mach. Learn..

[46] James G., Witten D., Hastie T., Tibshirani R. (2014). An Introduction to Statistical Learning: With Applications in R.

[47] Altman N.S. (1992). An introduction to kernel and nearest-neighbor nonparametric regression. Am. Stat..

[48] Sugeno M. (1985). Industrial Applications of Fuzzy Control.

[49] Rasmussen C.E., Williams C.K.I. (2005). Gaussian Processes for Machine Learning (Adaptive Computation and Machine Learning).

[50] Pedregosa F., Varoquaux G., Gramfort A., Michel V., Thirion B., Grisel O., Blondel M., Prettenhofer P., Weiss R., Dubourg V. (2011). Scikit-learn: Machine Learning in Python. J. Mach. Learn. Res..

[51] Fawcett T. (2006). An Introduction to ROC Analysis. Pattern Recognit. Lett..

[52] Sokolova M., Lapalme G. (2009). A systematic analysis of performance measures for classification tasks. Inf. Process. Manag..

[53] Trejos T., Corzo R., Subedi K., Almirall J. (2014). Characterization of toners and inkjets by laser ablation spectrochemical methods and Scanning Electron Microscopy-Energy Dispersive X-ray Spectroscopy. Spectrochim. Acta Part B At. Spectrosc..

[54] Aitken C. (1995). Statistical and the Evaluation of Evidence for Forensic Scientists.

[55] Kingma D.P., Ba J. (2014). Adam: A Method for Stochastic Optimization. arXiv.

[56] Chui C.K. (1992). An Introduction to Wavelets.

[57] Pořízka P., Klus J., Képeš E., Prochazka D., Hahn D.W., Kaiser J. (2018). On the utilization of principal component analysis in laser-induced breakdown spectroscopy data analysis, a review. Spectrochim. Acta.

