# Supplementary figures and images for: Application of Computational Intelligence Methods for the Automated Identification of Paper-Ink Samples Based on LIBS
Source: Sensors (Basel). 2018 Oct 29;18(11):3670. doi: 10.3390/s18113670 (PMC6263904; doi:10.3390/s18113670)

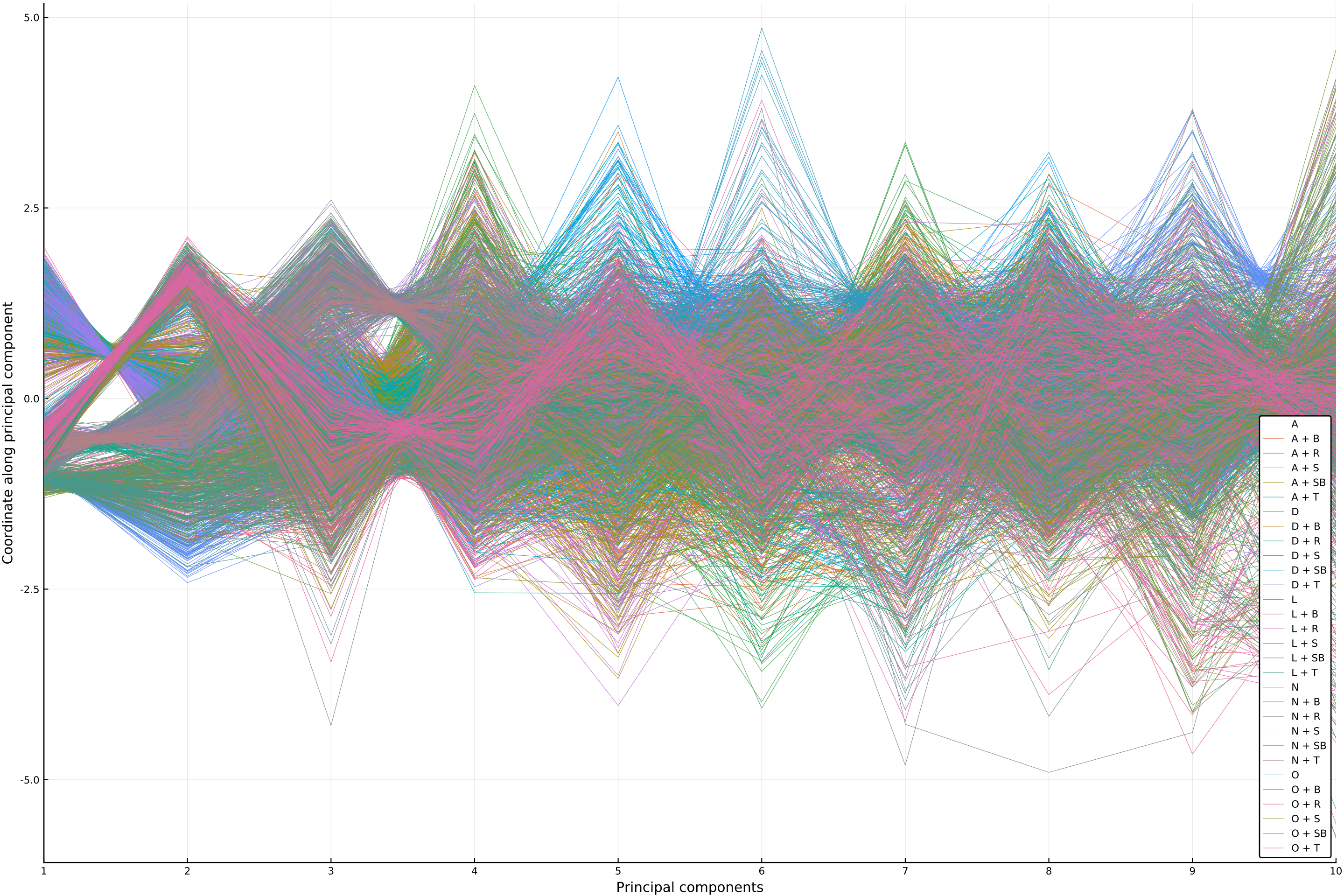

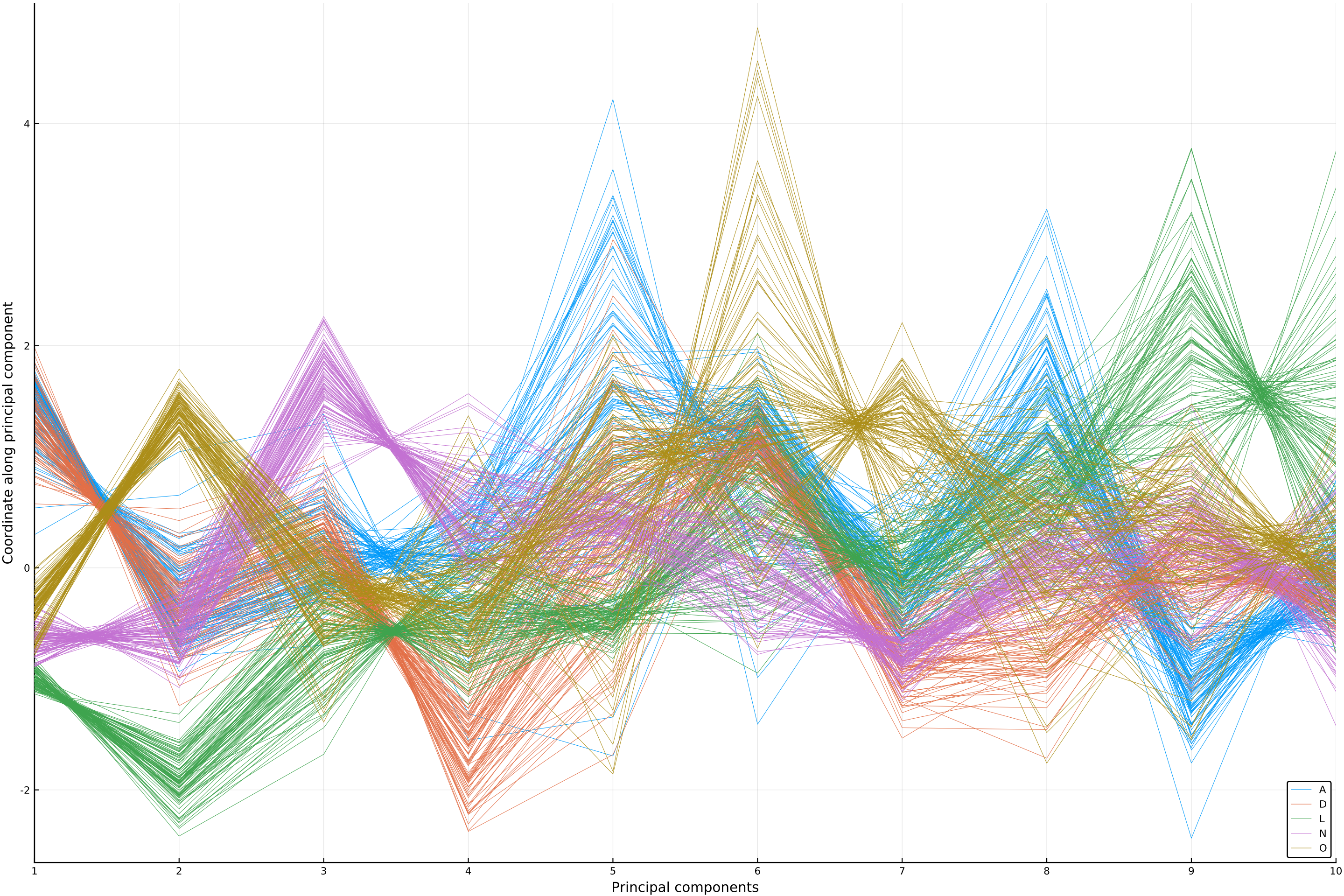

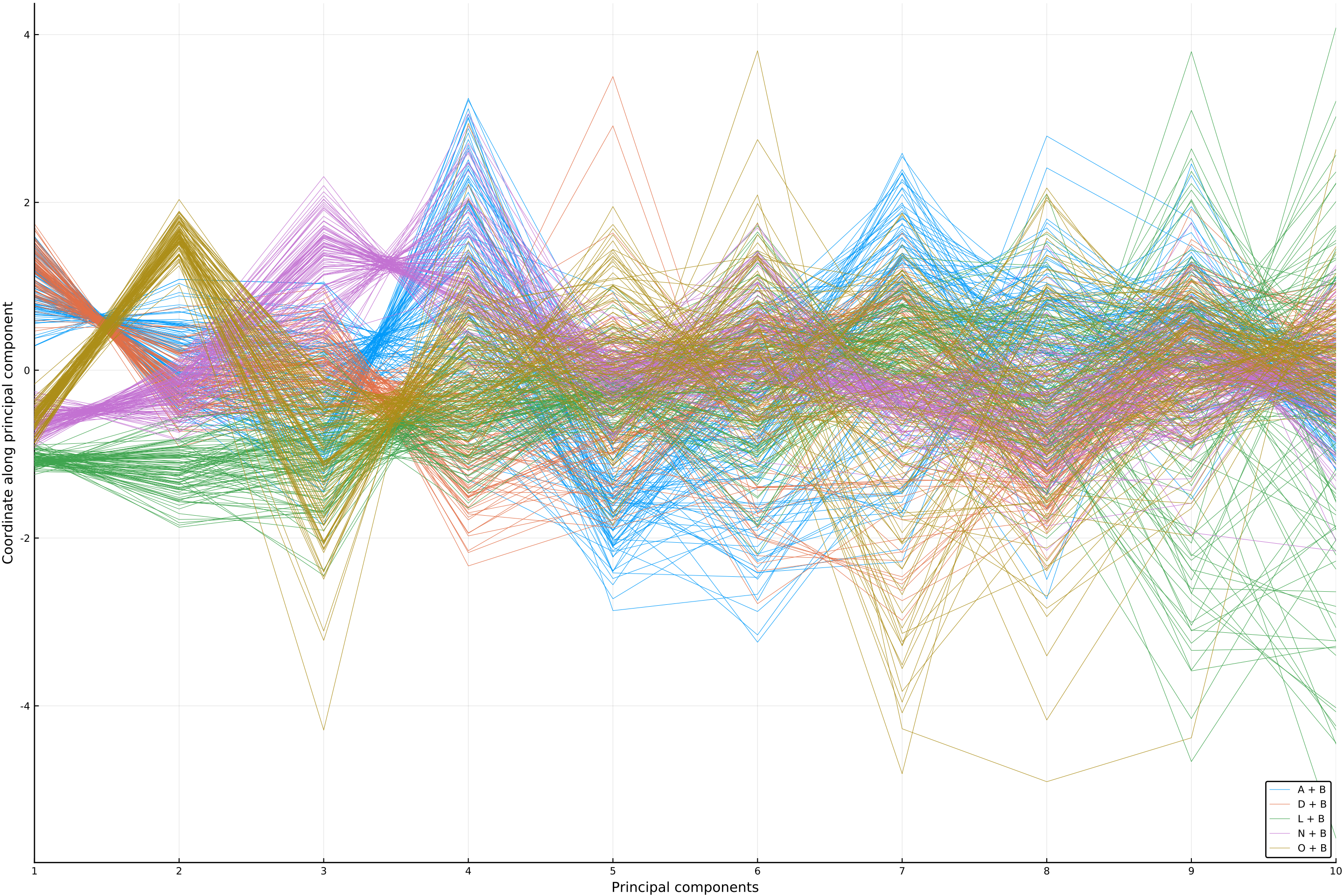

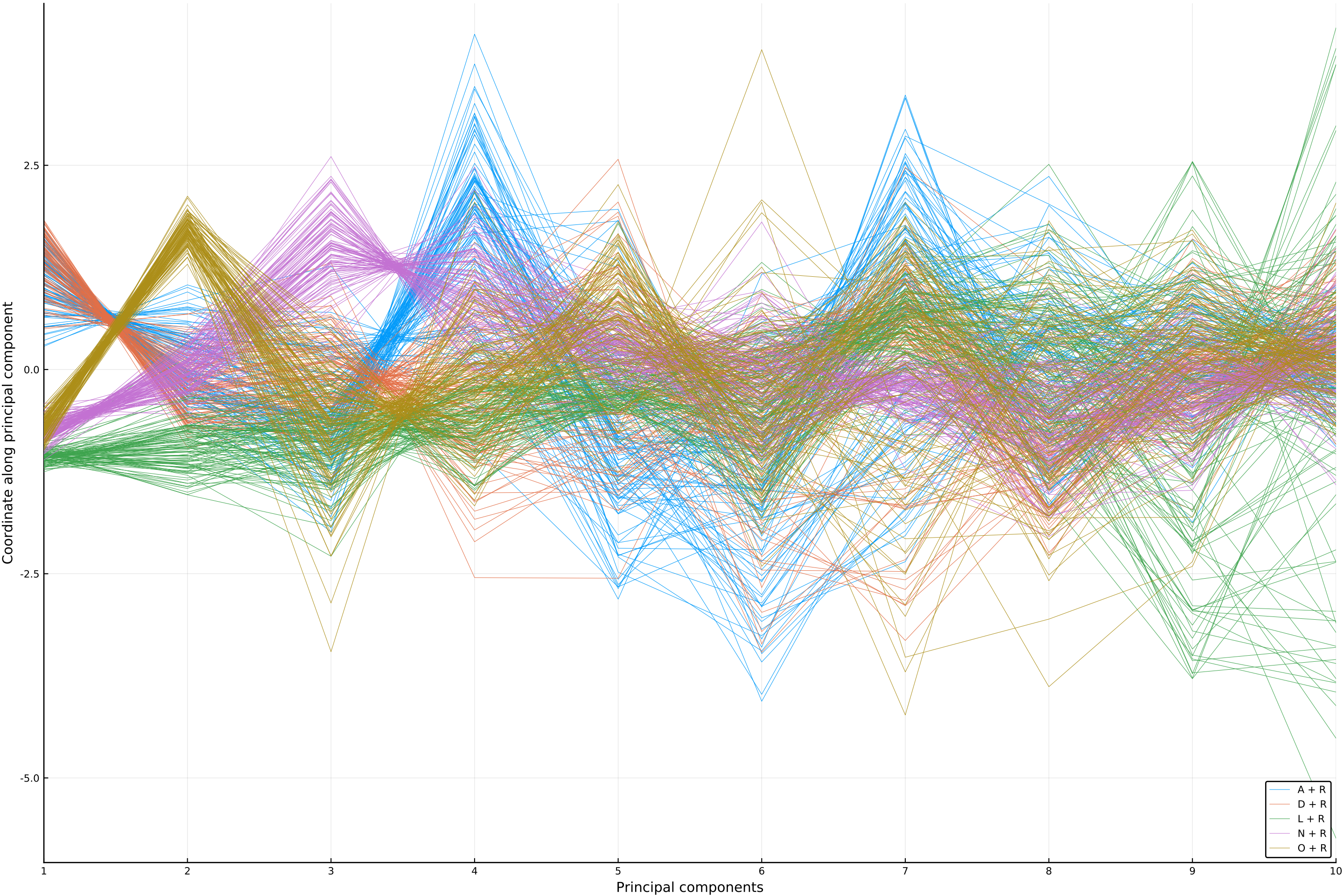

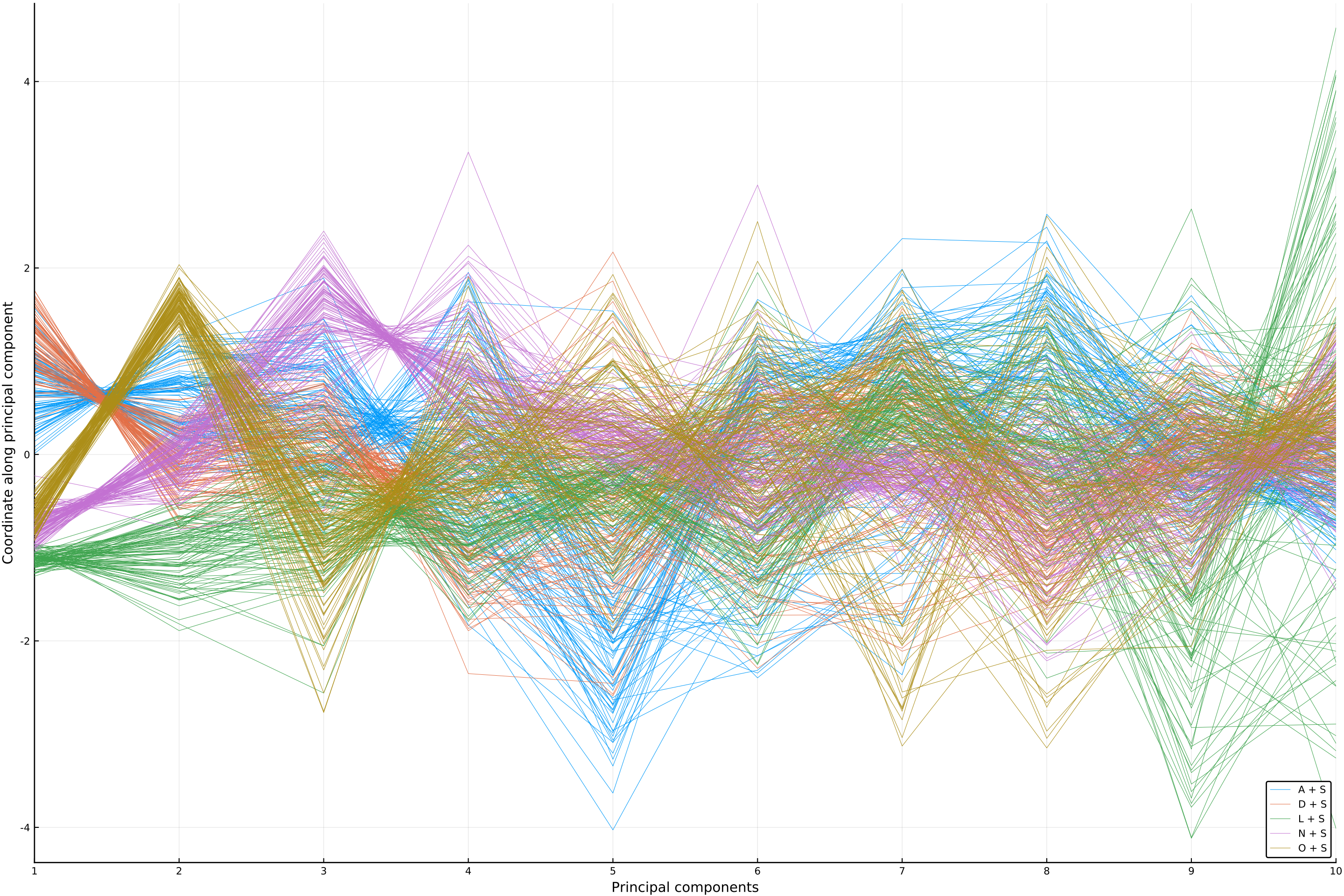

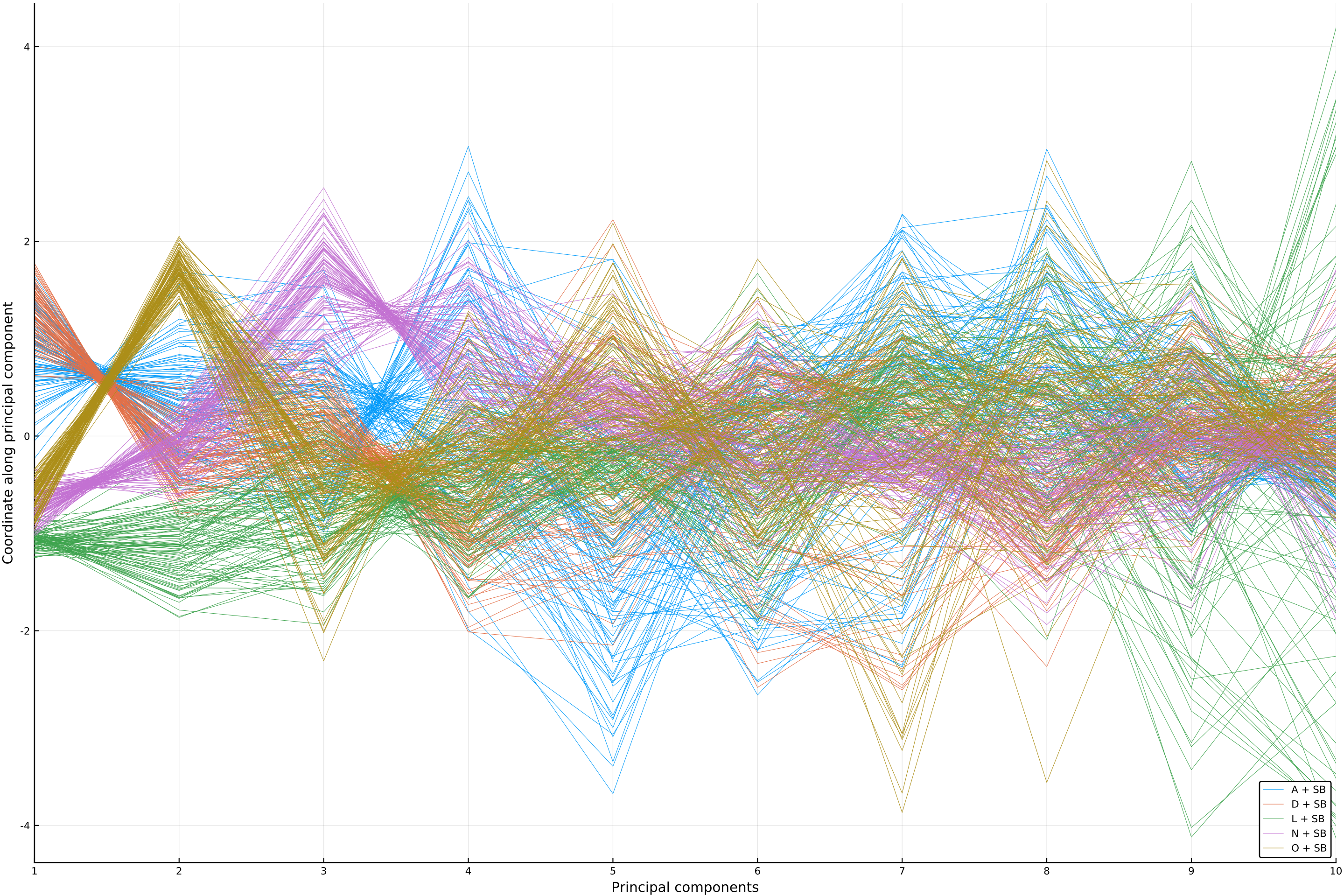

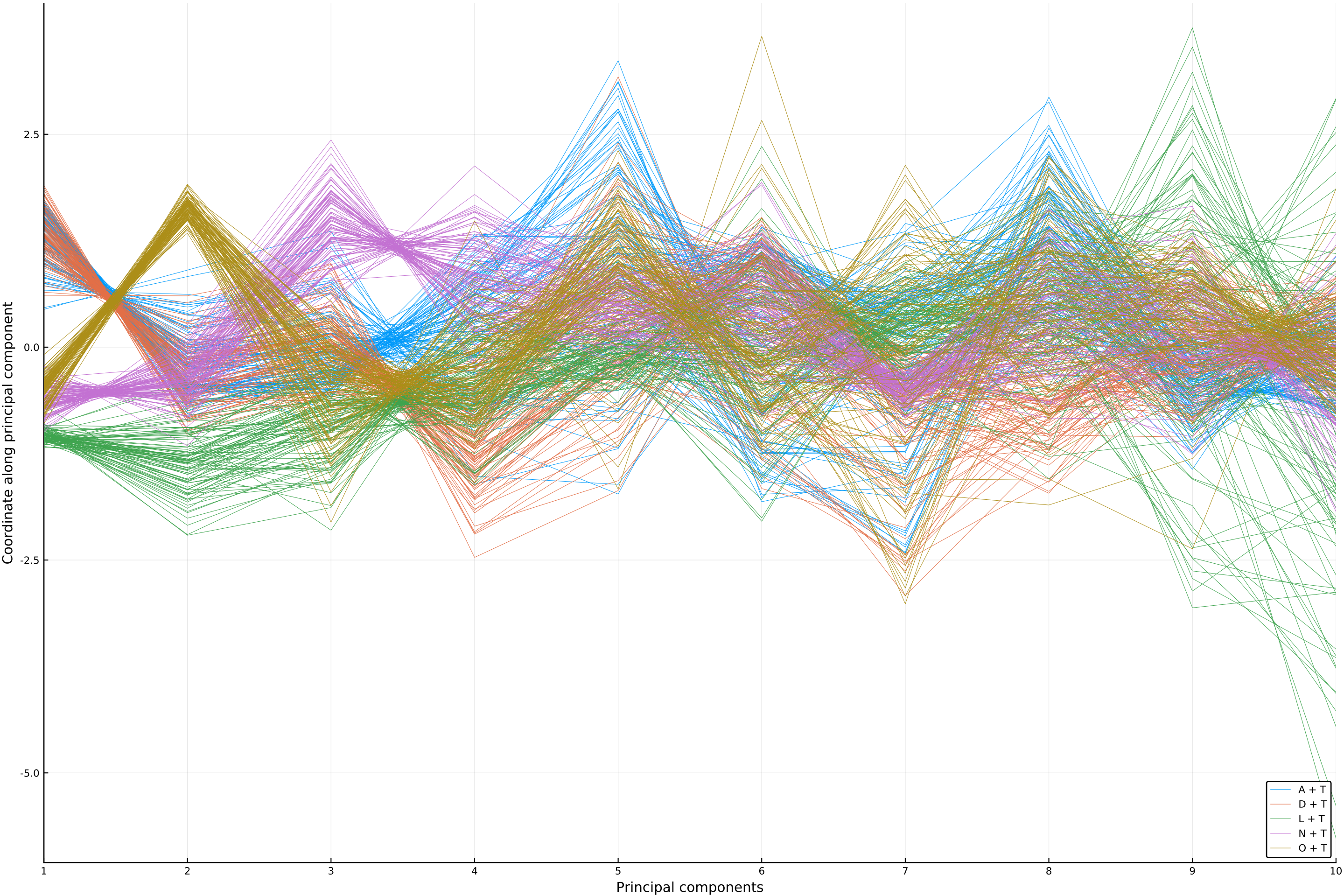

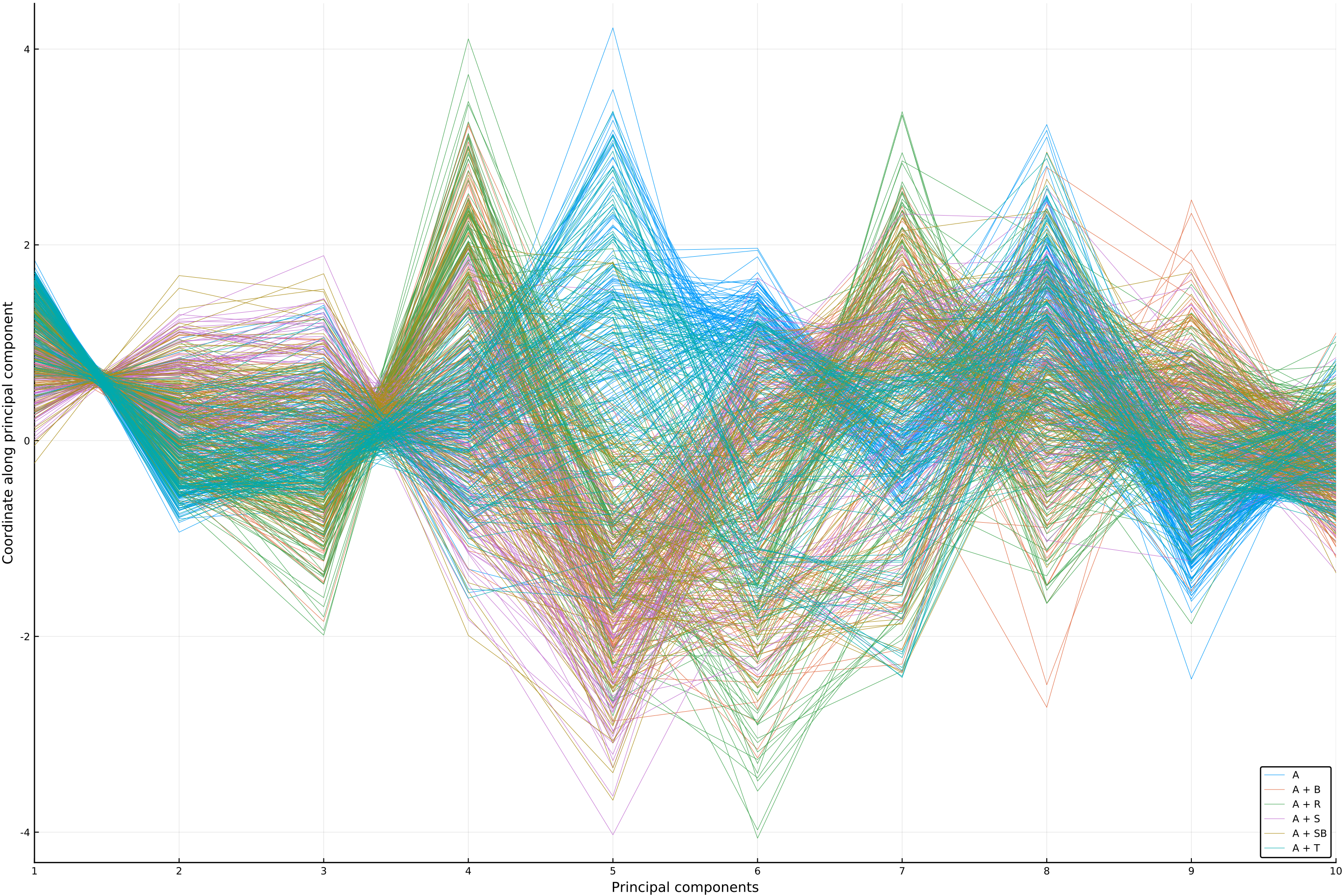

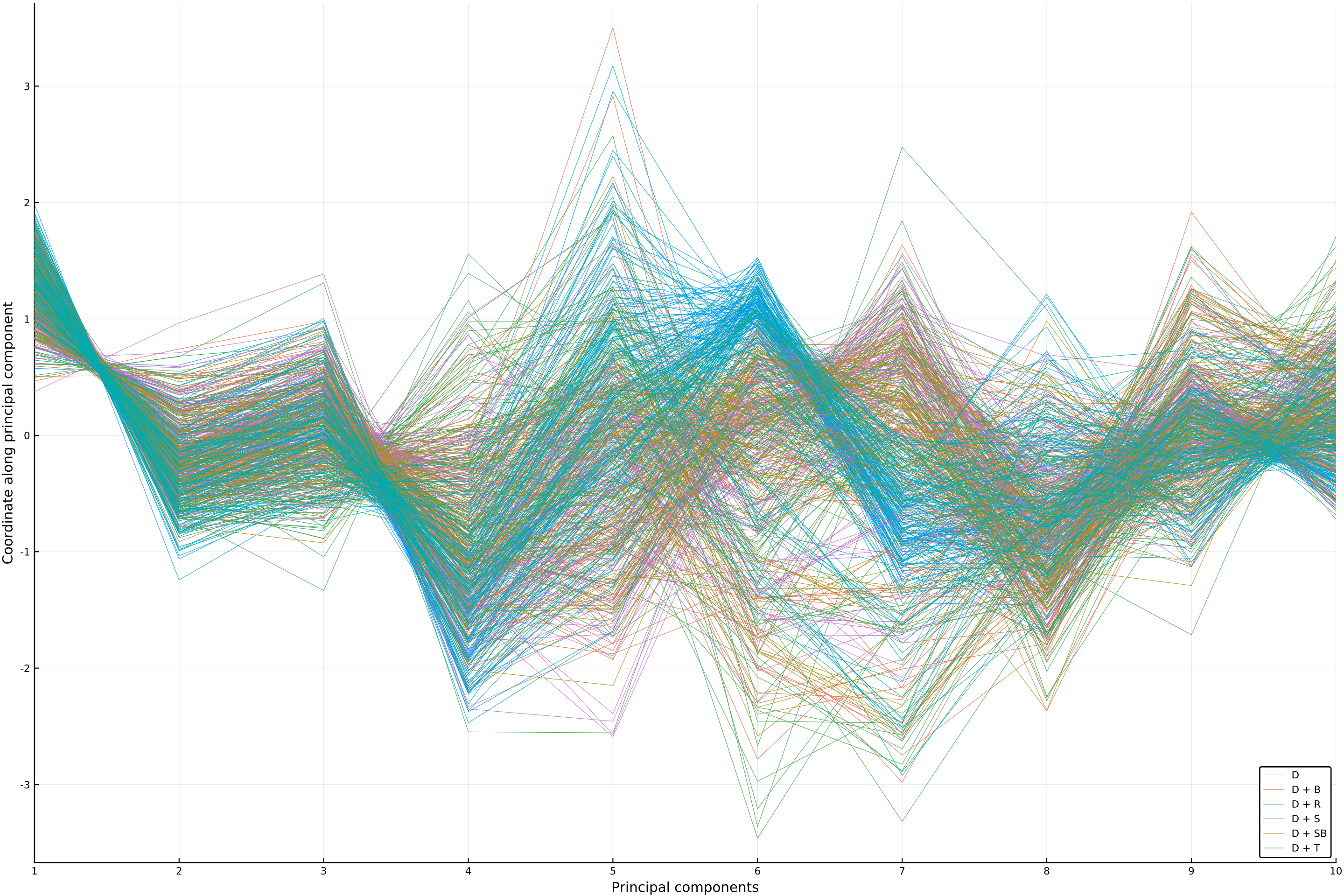

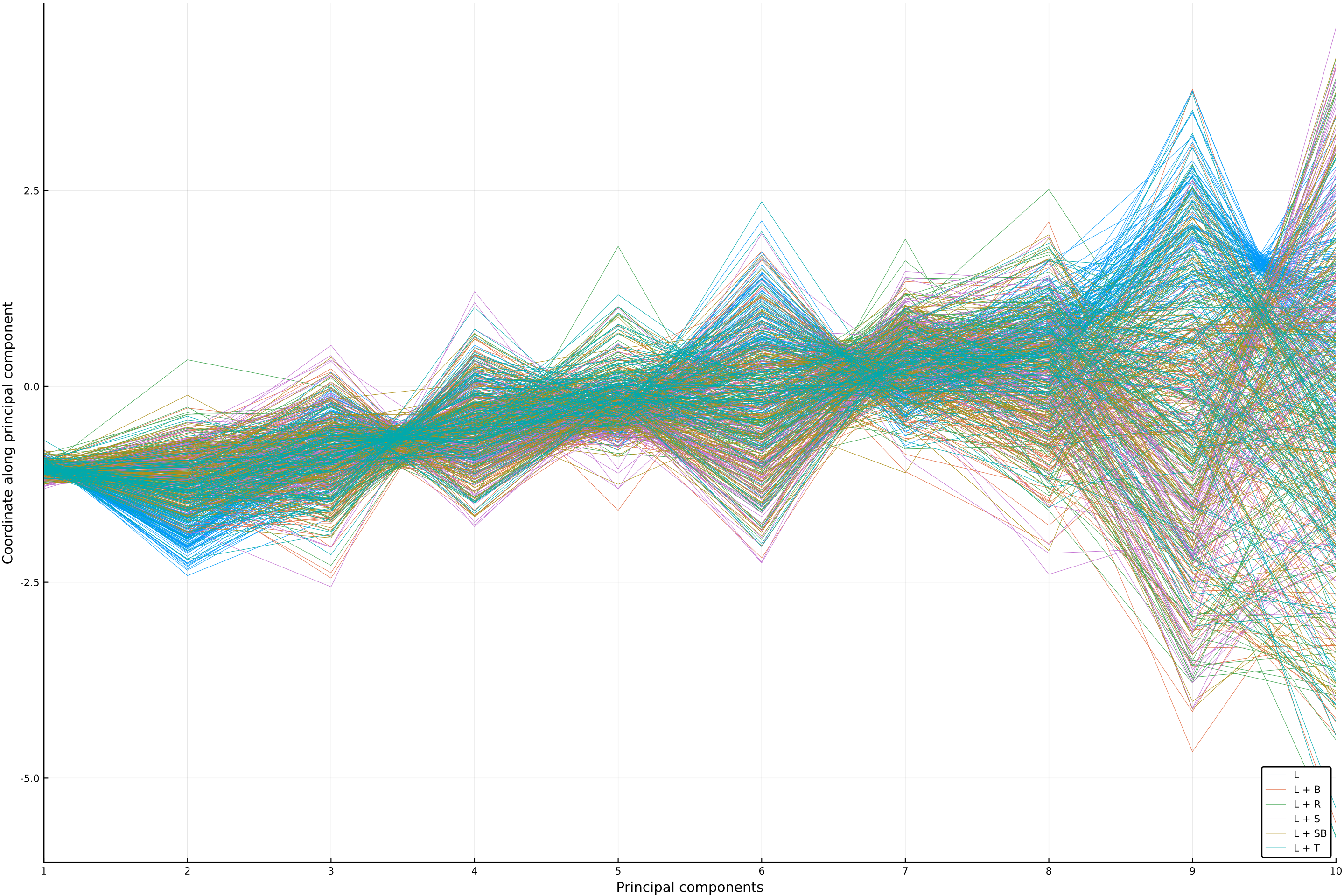

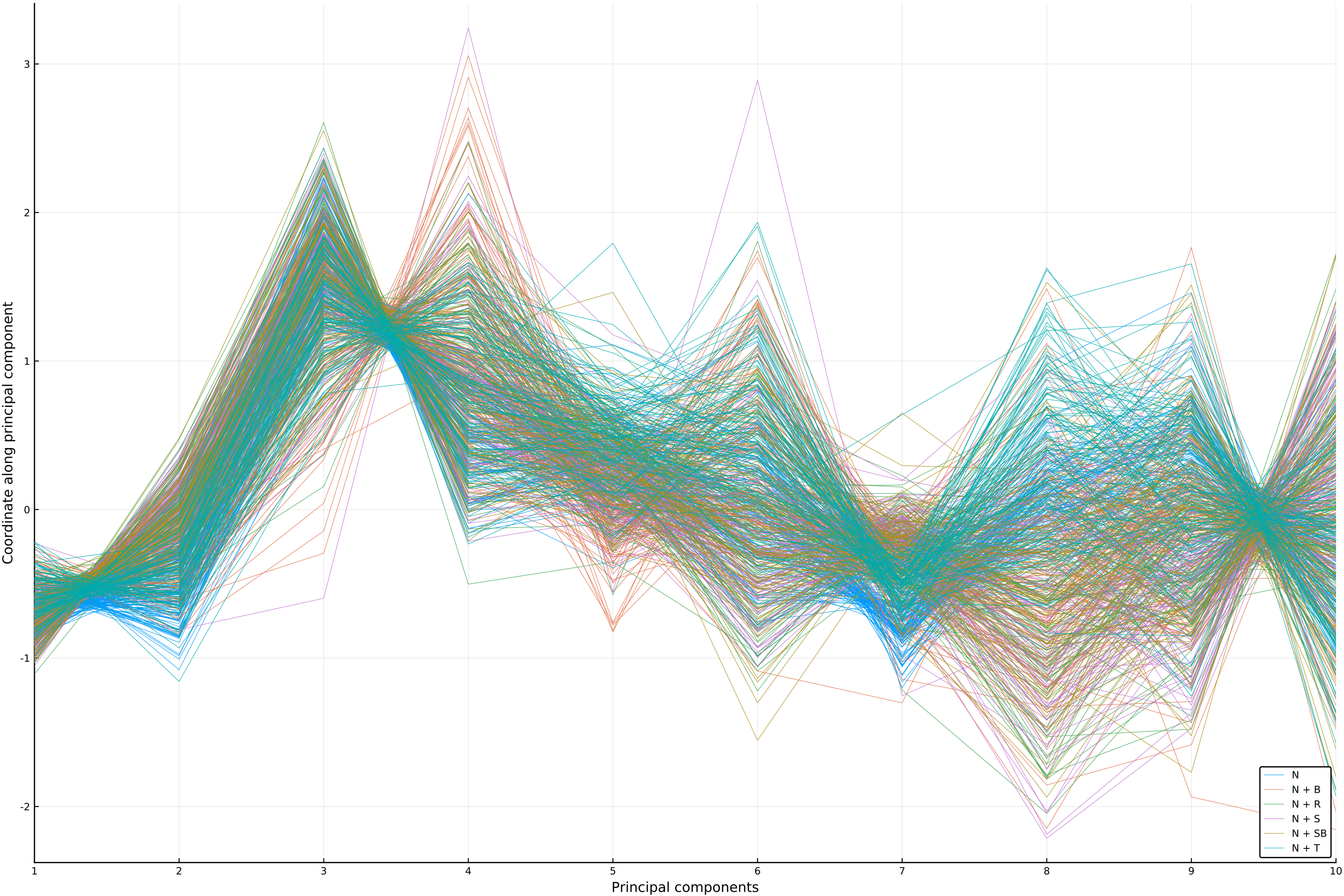

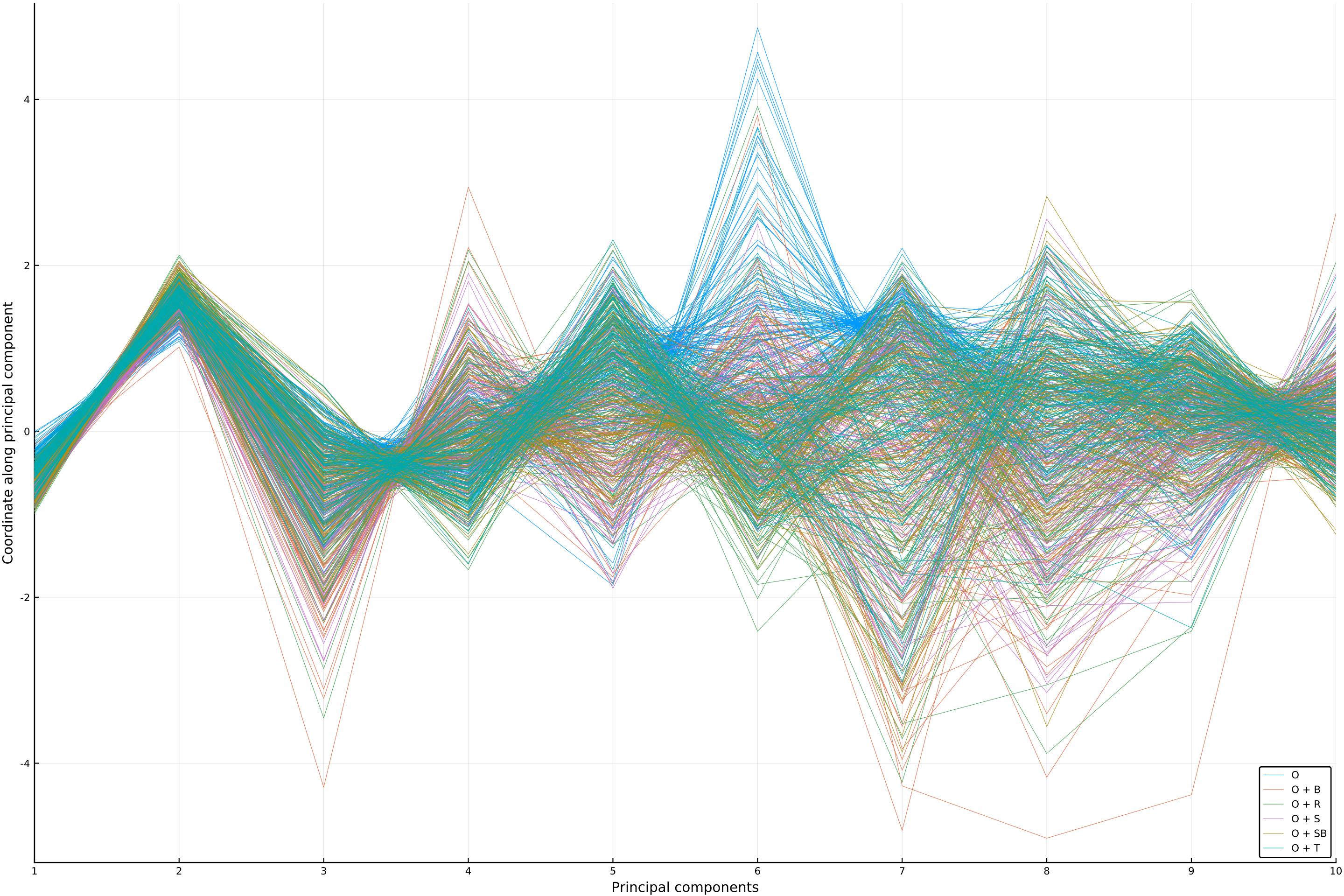

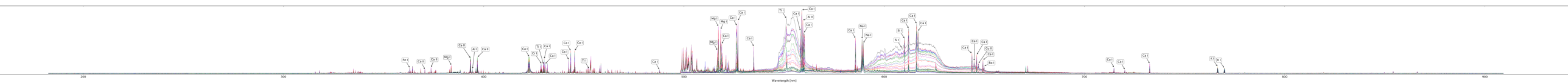

Supplement: Supplementary file 1 [file sensors-18-03670-s001.pdf]
